# Meteorological and environmental drivers of West Nile virus prevalence in *Culex pipiens* mosquitoes in Emilia-Romagna, Italy in 2013 to 2022

**DOI:** 10.1371/journal.ppat.1013753

**Published:** 2025-12-05

**Authors:** Victoria M. Cox, Katie Tiley, Roberto Rosa, Andrea Pugliese, Paola Angelini, Marco Carrieri, Samir Bhatt, Marco Tamba, Giovanni Marini, Mattia Calzolari, Ilaria Dorigatti

**Affiliations:** 1 MRC Centre for Global Infectious Disease Analysis, School of Public Health, Imperial College London, London, United Kingdom; 2 Centre for Mathematical Modelling of Infectious Diseases, The Department of Infectious Disease Epidemiology, Faculty of Epidemiology and Population Health, London School of Hygiene and Tropical Medicine, London, United Kingdom; 3 Center Agriculture Food Environment, University of Trento, San Michele all’Adige (TN), Italy; 4 Research and Innovation Centre, Fondazione Edmund Mach, San Michele all’Adige (TN), Italy; 5 Department of Mathematics, University of Trento, Trento, Italy; 6 Regional Health Authority of Emilia-Romagna, Bologna, Italy; 7 Centro Agricoltura Ambiente “Giorgio Nicoli”, Sanitary Entomology and Zoology Department, Crevalcore, Italy; 8 Section of Epidemiology, Department of Public Health, University of Copenhagen, Copenhagen, Denmark; 9 Istituto Zooprofilattico Sperimentale della Lombardia e dell’Emilia Romagana, Brescia, Italy; University of California Davis, UNITED STATES OF AMERICA

## Abstract

As West Nile Virus (WNV) is expanding its geographical range across Europe, there is an urgent need to characterise and better understand its transmission drivers to inform public health surveillance, disease control, and preparedness planning. We utilised 10 consecutive years of large-scale and fine-resolution WNV entomological field surveillance data from the Emilia-Romagna region in northern Italy, to evaluate the relationships between WNV infection rates in *Culex* mosquitoes and environmental and climatic conditions as well as WNV presence in the avian reservoir. We used fine-scale spatiotemporal regression models including non-linearities, to assess the drivers of presence and prevalence of WNV-positive mosquitoes. We validated the model estimates against reported cases of human WNV neuroinvasive disease in the region. We found evidence of established hotspots of mosquito WNV infection across multiple years. The presence of WNV in local birds was positively associated with presence and prevalence of WNV-positive mosquitoes (mean regression coefficients: 0.776 (95% CrI, 0.469, 1.08) and 0.226 (95% CrI, 0.053, 0.399) respectively), and the proportion of agricultural land use was positively associated with presence of WNV-positive mosquitoes (4.20 (95% CrI, 2.65, 5.75)). We identified a minimum temperature threshold around 13°C, below which mosquito WNV infection was reduced. Our findings provide evidence of the impact of temperature and environment on *Culex* populations and WNV infection dynamics at the local level, which were highly correlated with human case reports. The estimated role of the minimum temperature and the observed and projected increase in this variable under climate change suggest that WNV will continue to represent a risk for human and animal health in the region in future decades. Future work should focus on better understanding the mechanisms behind infection drivers, on the optimal implementation of surveillance and control activities around high-risk areas, and on the assessment of how specific land use practices could represent potential solutions to WNV infection.

## Author summary

West Nile virus (WNV) transmission in Europe is expanding, and the infection and disease burden is increasing across several countries. In this study we used ten consecutive years of entomological surveillance data from the Emilia-Romagna region in northern Italy, and fine-scale spatiotemporal regression models to evaluate the relationships between WNV infection rates of mosquitoes and the local environment and climate. We found that the minimum weekly temperature, WNV presence in local birds, and agricultural land use were key predictors of WNV infection rates in mosquitoes. In particular, we identified a minimum temperature threshold around 13°C, below which the WNV infection risk was reduced. The observed and projected increase in minimum temperatures across Europe and globally under climate change suggest that WNV will be an increasing risk for human and animal health. Model estimates correlated with the numbers of reported human neuroinvasive (WNND) disease cases, and suggest that the early onset of WNV infection in mosquitoes is associated with larger numbers of WNND cases in humans in the region. The data-driven model developed in this study lays the foundations for the development of forecasting and early-warning tools based on entomological WNV surveillance in the future.

## Introduction

West Nile virus (WNV) is a global health threat with widespread geographical distribution. It is maintained in a transmission cycle between *Culex* sp. mosquitoes, primarily *Culex pipiens* in Europe, and wild birds including hooded crows, jays, and magpies [1–4]. Humans and other mammals such as horses are infected with WNV following spill-over events, but they do not generate high enough viremia to infect biting mosquitoes and are therefore dead-end hosts [5], although human-to-human transmission can occur through infected blood and organ donation [6].

Six of the nine lineages of WNV are circulating in Europe, where the geographical range of WNV is rapidly expanding [7]. In Italy, WNV was first reported in horses in 1998 [8], with the first laboratory confirmed human case reported in 2008 [9], and WNV has been circulating in Emilia-Romagna since at least that year [10].

It is estimated that only ~20% of cases present symptoms such as fever, headaches, and joint pain and <1% develop severe neuroinvasive disease (WNND) which may be fatal [11]. Developing a WNV vaccine is a priority, and several candidates are under development [12,13]. Disease control currently relies on blood screening and vector control interventions triggered by entomological and veterinary surveillance. Organ, tissue (including blood), and cell products from donors in high transmission areas such as Emilia-Romagna are screened during WNV transmission seasons [6], which requires substantial resources and poses increasing economic costs to local and national public health agencies.

Laboratory studies have demonstrated associations between meteorological conditions and mosquito biology traits including development, feeding, survival, and mosquito-pathogen interactions [14–17]. Furthermore, previous studies where field data was analysed, demonstrated the influence of environmental temperatures on mosquito population dynamics and distribution; for example, high temperatures in early spring increased *Culex* abundance in Piedmont, Italy [18–20]. Relationships between environmental temperatures and equine epidemics and human WNV outbreaks have also been demonstrated [20–28]. Despite recent advances in our understanding of the temperature-dependency of ecological processes underpinning mosquito population dynamics, capturing the spatiotemporal patterns of WNV transmission and infection remains challenging, due to the sparsity of high resolution entomological WNV prevalence data, the limited number of WNND human cases reported through surveillance, and limited insights into local risk-factors of WNV. It has previously been shown that mosquito abundance was not strictly correlated with, nor a limiting factor for, WNV circulation in Northern Italy in 2018, whereas WNV infection rates in mosquito populations were correlated with the incidence of human cases [29]. Therefore, in order to better understand the drivers of WNV transmission in the context of public health, it is important to characterise the impact of climate and environment on infection rates, or prevalence, in mosquito populations.

The aims of this study were to (1) estimate the prevalence of WNV from high-resolution entomological surveillance data collected at the same 70 locations in Emilia-Romagna from 2013 to 2022, (2) develop and apply multivariable spatiotemporal models to real-world estimates of mosquito WNV prevalences at fine spatial resolution, (3) provide new insights into the risk factors of mosquito WNV transmission in Emilia-Romagna, which may aid future mechanistic model design and inform disease control and the optimal use of local resources, and (4) assess to what extent estimates of mosquito WNV risk are correlated with reports of human WNND cases.

## Methods

### Data

#### Mosquito WNV surveillance.

**Fig 1:**
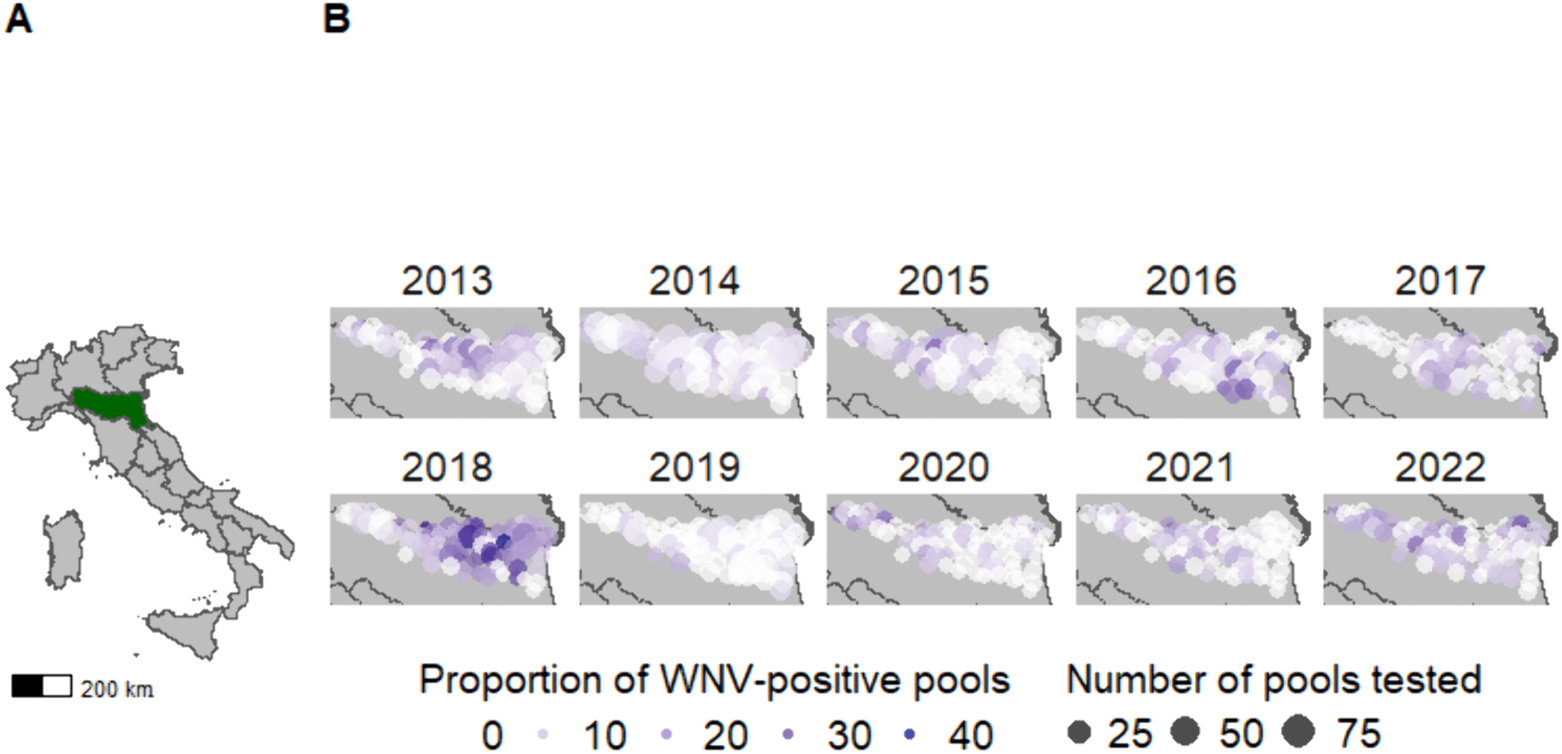
West Nile Virus positive mosquito pools across the Emilia-Romagna region between 2013 and 2022. The Emilia-Romagna region in Italy is highlighted in dark green (A). Across Emilia-Romagna, annual maps show the total number of mosquito pools sampled at each trap by the point size, and the proportion (%) of WNV-positive pools is shown by the navy colour scale (B). The shapefile used to generate the base layer maps was obtained GADM (https://gadm.org/download_country.html and https://gadm.org/license.html).

Mosquitoes were captured in a surveillance network of traps in Emilia-Romagna, Italy, composed by more than 90 entomological traps (CO_2_ and Gravid traps), 70 of which remained in the same site throughout the study period. Data from the 70 traps were used in this study (Fig 1). Traps were activated for each transmission season from approximately June to October during 2013–2022 (19 weeks per year on average, range 17–24), and were monitored every two weeks from 17:00 until 9:00 the following morning. Mosquitoes were identified morphologically to the species level [30,31], combined into pools of up to 200 specimens (average pool size = 113), and screened for WNV at the pool level using two different PCR protocols [32,33]. As the primary vector species for WNV in the region, only *Culex pipiens* were included in this analysis. The proportion of WNV-positive pools at each location per year is shown in Fig 1.

#### Avian WNV surveillance.

Active WNV surveillance in birds was performed at provincial level (Nomenclature of Territorial Units for Statistics, NUTS 3) in Emilia-Romagna each transmission season, from approximately May to November for the years 2013–2022, specifically targeting *Corvus cornix* (Hooded Crow), *Garrulus glandarius* (Eurasian Jay), and *Pica pica* (Magpie), taking advantage of containment plans for these species that are considered agriculture pests. Each province was divided into sectors of 1200–1600 km^2^ and at least 20 birds per sector were collected each month, using walk-in traps or by shooting. The heart, spleen, kidneys, and brain of each bird were pooled and tested for WNV RNA using RT-PCRs [32–34] (S1 Fig). Since the specific hunting routes within provinces were unknown, the culling location was defined as the centroid of each province where the birds were culled, using administrative boundaries described in the shapefiles obtained from Istituto Nazionale di Statistica (https://www.istat.it/it/archivio/222527). Avian WNV presence was determined at each trap if PCR positive birds were collected within a 20 km radius to the trap (S2 Fig). All 70 traps were within 20 km range of at least one province centroid (mean number of centroids within range per trap = 10.36, median = 10, IQR = 7–13). Because the sensitivity of avian surveillance is low and birds are territorial on the modelled scale, we assumed avian WNV presence at the trap level every year from the first week in which avian WNV presence was detected to the end of the transmission season. A sensitivity analysis relaxing the assumption of avian WNV presence from the first detection week was performed (Supplementary Material).

#### Human infection data.

The total weekly reported number of WNV neuroinvasive disease cases (WNND) in Emilia-Romagna between 2013 and 2022 was provided by Regional Health Authority of Emilia-Romagna.

#### Meteorological data.

Daily meteorological data from 2013 to 2022 was provided by the Regional Environmental Agency (ARPAE Emilia-Romagna) according to a high-resolution 5km x 5km gridded interpolated dataset [35]. This included average, minimum, and maximum air temperature (^o^C), average relative humidity (%), cumulative precipitation (mm), hours of leaf wetness (hr), average global solar radiation (MJ m-2), and evapotranspiration (mm) calculated using the Penman-Monteith formulae which takes air temperature, solar radiation, humidity, and wind speed as input [35]. The daily data was extracted for each trap location over the timeseries and summarised at the weekly level to produce cumulative precipitation and mean averages of the other variables, as well as the absolute minima and maxima temperature of each week (S3 Fig). The variables were lagged by 1, 2, and 3 weeks, and we also considered averages over sequential weeks including the previous 1, 2, and 3 weeks.

#### Land use and elevation data.

Different types of land use were identified in a 3 km buffer zone around each trap location, categorised according to 2012 CORINE land cover (CLC) level I classes: CLC1 artificial surfaces, CLC2 agricultural areas, CLC3 forest and seminatural areas, CLC4 wetlands and CLC5 water bodies. CLC were extracted from shapefiles provided by the Institute for Protection and Environmental Research (ISPRA) [36] using the free software Qgis [37]. We estimated the proportion of each land cover class in the 3 km buffer around each trap (S4 and S5 Figs). We also categorised the land use around each trap according to CLC level III classes. The top 5 CLC level III categories were: CLC211 non-irrigated arable land (74% of land around all the traps), CLC242 complex cultivation patterns (8.5%), CLC112 discontinuous urban fabric (3.9%), uncharacterised (2.3%), and industrial and commercial units CLC121 (2.0%). The geolocation coordinates for each trap were used to collate the elevation [38] (S4 Fig).

### Estimating prevalence of WNV-positive mosquitoes

We estimated the prevalence of WNV-positive mosquitoes from the pooled entomological data after PCR testing for WNV presence, using the *PooledInfRate* R package [39]. We calculated point estimates and confidence intervals of the prevalence from pooled binary data made up of differing pool sizes, using the bias-corrected maximum liklihood estimate (MLE) and Firth’s correction [40].

At each trap and collection time point, trapped mosquitoes were grouped into $n_{k}$ number of pools of sizes $m_{k}$ with k number of different pool sizes, k= 1, ..., d (the total mosquito count equals $\sum_{k=\text{1}}^{d}n_{k}m_{k}$). The number of positive pools of size $m_{k}$ ($x_{k}$) was assumed to follow a binomial distribution: $x_{k}\;\sim \;Binomial(n_{k},\;\text{1}-(\text{1}-p{)}^{m_{k}})$, where p is the prevalence. The log-likelihood, logLik(p;x) was calculated over all pool sizes following Equation 1. In summary, Equation 1 was used to estimate the prevalence of WNV-positive mosquitoes at each trap and week (i.e., the proportion of infected mosquitoes out of all those sampled and tested, which can be thought of as a proxy for abundance).


$$logLik(p;x)=\;\sum_{k=\text{1}}^{d}x_{k}\;log\left[\left(\text{1}-(\text{1}-p{)}^{m_{k}}\right)\right]+log(\text{1}-p)\sum_{k=\text{1}}^{d}{(m}_{k}{(n}_{k}-\;x_{k}))$$ (1)

### Estimating Vector Index

Vector Index (VI) is the prevalence of WNV-positive mosquitoes multiplied by the number of mosquitoes tested, per trap and time point. We estimated VI and performed a sensitivity analysis fitting the models (which are described below for the prevalence of WNV-positive mosquitoes) to the VI as the response variable (see Supplementary Material).

### Spatiotemporal regression model

Spatiotemporal models were fitted to the prevalence point estimates at the trap and week level. The prevalence estimates at trap i and week t ($p_{i,t}$) were highly zero-inflated (S6 Fig), thus hurdle models were fitted with a Bernoulli likelihood for the occurrence data ($z_{i,t}$) at trap i and week t, and a gamma likelihood for the log transformed $p_{i,t}$ ($y_{i,t}$) at occurrence (Equation 2).


$$z_{i,t}=\;\left\{ \;\;\;\begin{array}{c} \text{1},\;if\;p_{i,t}>\text{0}\;\\ \text{0},\;if\;p_{i,t}=\;\text{0}\; \end{array}\right.\;$$

$$y_{i,t}=\;log(p_{i,t}),\;where\;p_{i,t}>\text{0}$$ (2)

Presence and prevalence of WNV-positive mosquitoes were assumed to be continuous processes (Gaussian field (GFs)) across the region, determined by intra- and inter-annual temporal relationships and spatial correlation. These assumptions were justified in preliminary analyses which assessed the spatial autocorrelation in the entomological data (see Supplementary Material).

We developed spatiotemporal models in R-INLA using the stochastic partial differential equations (SPDE) approach [41–43], which represents the GFs as discrete processes called fields (see Supplementary Material). We performed an initial analysis comparing model alternatives with different fields for the baseline spatiotemporal model (see Supplementary Material) using the Watanabe-Akaike Information Criterion (WAIC) which is a measure of prediction error [44]. The best baseline model included annual fields for z where the fields per year were related to sequential years through an autoregressive function of order 1, and a field for y assuming the fields per year were independent.

To demonstrate the utility of the methodologies presented in this study, and specifically the effect of accounting for spatiotemporal autocorrelation (through the spatiotemporal fields) on the model predictive performance, we performed a sensitivity analysis where we developed the models starting with a baseline model without the spatiotemporal fields (see Supplementary Material).

### Variable selection

To assess whether meteorological variables should be included in the regression models as linear or non-linear effects, we performed an initial analysis where each variable was categorised according to its 10^th^ to 90^th^ percentiles and added to the baseline spatiotemporal model as a random effect categorical variable. The linearity of the regression coefficient pattern over the percentiles was assessed (see Supplementary Material).

The meteorological, land use (CLC1 classes), and avian WNV presence variables were added to the baseline spatiotemporal model one at a time in a univariable analysis where prevalence was modelled following Equation 3. Intercepts are represented by α, $\mu_{z,i,a}$ is the field for z for each year a, $\mu_{y,i,a}$ is the field for y for each year a, and $X_{i,t}$ represents the variable with regression coefficients β (separate β for the same $X_{i,t}$ in the predictor for $z_{i,\;t}$ and $y_{i,t}$, denoted $\beta_{z}$ and $\beta_{y}$) when variables were linearly associated with presence or prevalence. For variables that were non-linearly associated with prevalence, we included them as random walks of order 2,${\;\psi_{z}}_{i,t}$ and ${\;\psi_{y}}_{i,t}$ (see Supplementary Material). In the main analysis the land use variables were categorised using CLC level I classes, and in an additionally analysis we fit univariable models using land use variables categorised with CLC level III classes.


$$z_{i,\;t}=\;\alpha_{z}+A+\;\mu_{z,i,a}$$

$$y_{i,t}\;={\;\alpha }_{y}+B+\;\mu_{y,i,a}$$

$$\text{A}=\left\{ \;\;\begin{array}{c} \;\;\;\;\;\;\;\;\;{\beta_{z}X}_{i,t},\text{ for linear variables}\\ {\;\psi_{z}}_{i,t},\text{ for non}-\text{linear variables} \end{array}\right.\text{ B}=\left\{ \;\;\begin{array}{c} \;\;\;\;\;\;\;\;\;\;\;{\beta_{y}X}_{i,t},\text{ for linear variables}\\ {\;\psi_{y}}_{i,t},\text{ for non}-\text{linear variables} \end{array}\right.\;$$ (3)

The WAIC scores of the univariable models were compared to identify the best predictor of the presence and prevalence of WNV-positive mosquitoes, i.e., the variable in the univariable model with the lowest WAIC score.

A step-wise forward selection approach was used to build a multivariable model shown in Equation 4. The variables were added one at a time; either fixed-effect linear variables $\left(X_{i,t}\right)$ or non-linear variables which were included as random effects (${\psi_{z}}_{i,t}$ and ${\psi_{y}}_{i,t}$). The total number of linear variables added after all the selection steps was represented by l, and the regression coefficients for each $X_{i,t}$ ($X_{{\text{1}}_{i,\;t}}$ to $X_{l_{i,\;t}}$) were represented by$\;\beta_{z\text{1}}$ to $\beta_{zl}$ and $\beta_{y\text{1}}$ to $\beta_{yl}$. The total number of non-linear variables added was represented by n. At each model building step 1:(l+n), single variables were added to the model and included if: (1) they improved the WAIC score more than the other variables tested in that step, (2) the WAIC improvement compared to the previous step was > 4, (3) the variable was not collinear with any variables already included in the model from previous steps (as determined by a Pearson’s correlation coefficient > |0.6|), and (4) the variable was significantly associated with the response variable (the association was considered significant for linear variables if the 95% CrI of the β coefficients did not cross zero, and for non-linear variables if the 95% CrI of the random walk coefficients for all spine points did not overlap) (S2 and S3 Tables).


$$z_{i,\;t}\;=\;\alpha_{z}+\beta_{z\text{1}}X_{{\text{1}}_{i,t}}\ldots \beta_{zl}X_{l_{i,t}}+\;{\psi_{z\text{1}}}_{i,t}\ldots {\psi_{zn}}_{i,t}+\;\mu_{z,i,a}$$

$$y_{i,t}\;={\;\alpha }_{y}+\beta_{y\text{1}}X_{{\text{1}}_{i,t}}\;\ldots \beta_{yl}X_{l_{i,t}}+{\psi_{y\text{1}}}_{i,t}\ldots {\psi_{yn}}_{i,t}+\;\mu_{y,i,a}$$ (4)

### Assessment of model fit

To assess the performance of the hurdle model, we estimated the area under the curve (AUC) of the Receiver Operating Characteristic (ROC) curve using the *pROC* R package [45]. The ROC simultaneously assesses the specificity and sensitivity of the binary classifier. We estimated the mean absolute error (MAE) in the estimated prevalence at different spatial and temporal aggregations, which is defined as the sum of the absolute value of the difference between the estimated prevalence and the observed prevalence, divided by the number of estimates. We computed (1) the overall MAE which is the MAE averaged over space and time, (2) the MAE for each trap location averaged over time, and (3) the MAE per week averaged over all trap locations. The WAIC, overall MAE (for prevalence), and AUC (for presence) were compared between the baseline models and the final multivariable models.

### Assessment of the correlation between model output and reported human cases

For each year, the following metrics were calculated from the model-estimated risk of WNV-positive mosquitoes (a composite of the estimated occurrence, $z_{i,\;t}$, and prevalence, $y_{i,\;t}$) averaged over all traps: (1M) week with the highest risk (peak week), (2M) peak week after each timeseries was smoothed using LOESS regression, (3M) risk at the peak week, and (4M) risk at the smoothed peak week. For each year, the following metrics were calculated from the weekly number of reported human WNND cases in Emilia-Romagna: (1H) the first week with a reported case, (2H) week with the highest number of reported cases (peak week), (3H) peak week after the human case timeseries was smoothed using LOESS regression, (4H) cases at the peak week, and (5H) total number of cases in the season.

In addition, the timeseries of the model estimates and of the human WNND cases were smoothed and cross-correlation was performed using the *ccf* function in R. The correlation between the timeseries was assessed at all lags between 0- and 6 weeks.

## Results

Over 1.6 million *Culex pipiens* mosquitoes were sampled across the 70 traps during field-surveillance between 2013 and 2022. The greatest proportion of WNV-positive pools was observed in 2018 (Table 1) and the inter-annual patterns of prevalence of WNV-positive mosquitoes showed reduced infection in the seasons in 2019–2021 following the large 2018 season. Intra-annually, we observed prevalence of WNV-positive mosquitoes generally peaked in August each year (mean 33^rd^ week of the year, range 28^th^ to 36^th^). The spatial clustering of presence of WNV-positive mosquito pools changed each year (Fig 1), as did the sites with the greatest number of weeks where avian WNV presence was recorded (S2 Fig). There were significant positive associations between avian WNV presence and both presence and prevalence of WNV-positive mosquitoes in the univariable and final models (regression coefficients in the final multivariable model equal to 0.767 (95% CrI: 0.449, 1.08) and 0.248 (95% CrI: 0.073, 0.423) for presence and prevalence respectively). A sensitivity analysis showed that models including avian WNV presence where potential false negatives in the avian surveillance data were corrected for (as reported above), performed better than models including avian data without the correction. Aside from avian WNV presence, the majority of the tested predictors influenced the probability of presence of WNV-positive mosquito pools but were not significantly associated with the prevalence of WNV-positive mosquito pools. This pattern was observed also in the sensitivity analysis where we did not assume WNV presence in the avian hosts after its first detection.

**Table 1:** Mosquito pool characteristics.

Year	Number of pools tested	Number of specimens tested	WNV-positive pools	Mean pool size (IQR)
2013	1,557	227,926	92 (5·9%)	146 (72–200)
2014	2,555	221,208	84 (3·3%)	87 (25–200)
2015	1,569	161,220	59 (3·8%)	103 (50–200)
2016	1,663	173,908	84 (5·1%)	105 (50–200)
2017	986	114,321	42 (4·3%)	116 (50–200)
2018	1,164	166,347	147 (12·6%)	143 (65–200)
2019	1,790	270,981	22 (1·2%)	151 (91–200)
2020	1,021	99,729	34 (3·3%)	98 (26–200)
2021	1,111	112,379	37 (3·3%)	101 (24–200)
2022	1,028	91,401	64 (6·2%)	89 (18–200)

Total number of pools tested for West Nile virus (WNV), total number of *Culex pipiens* mosquitoes captured in each year, number of WNV-positive pools, and mean and interquartile range (IQR) of pool size by year.

**Fig 2:**
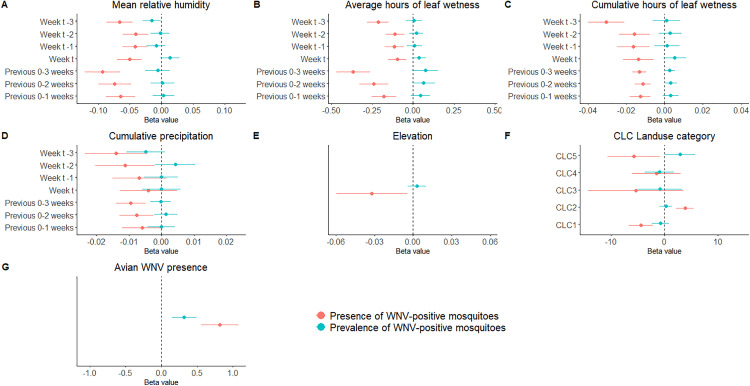
Univariable model beta coefficients for variables included as linear fixed effects. For the meteorological variables, the lag the variable was calculated over is shown on the y-axis. A dashed line is shown for beta values equal to zero to separate variables which were positively or negatively associated with presence of WNV-positive mosquitoes (red) or prevalence of WNV-positive mosquitoes (blue).

In the univariable analysis we observed significant positive associations between presence of WNV-positive mosquitoes and the proportion of CLC2 (agricultural land use), which was also included in the final multivariable model (regression coefficient = 4.43 (95% CrI: 2.55, 6.30)), and negative associations between presence of WNV-positive mosquitoes and the proportion of CLC5 and CLC1 (water bodies and artificial land use) (Fig 2). In the additional analysis where we fitted univariable models with CLC level III land use variables, discontinuous urban fabric (CLC112) was significantly negatively associated with both presence and prevalence of WNV-positive mosquitoes. Industrial and commercial units (CLC121), road and rail networks (CLC122), green urban areas (CLC141), continuous urban fabric (CLC111), salt marshes (CLC421), coastal lagoons (CLC521), and seas/ocean (CLC523) were negatively associated with presence of WNV-positive mosquitoes, while non-irrigated arable land (CLC211) was positively associated with presence of WNV-positive mosquitoes.

**Fig 3:**
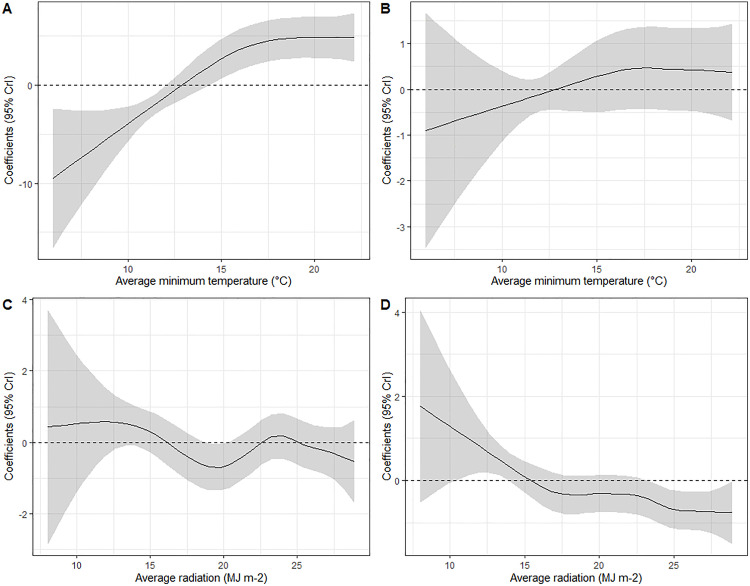
Non-linear relationships between meteorological variables and presence and prevalence of WNV-positive mosquitoes in the final multivariable models. Random walk coefficients for weekly minimum temperature averaged over the previous 0 to 3 weeks associated with presence of WNV-positive mosquitoes (A) and prevalence of WNV-positive mosquitoes (B), and weekly solar radiation averaged over the previous 0 to 1 weeks for presence (C) and prevalence (D). A dashed line is shown for coefficients equal to zero to separate values of the variables which were positively or negatively associated with the response variables.

The linear meteorological variables in the univariable analysis (relative humidity, precipitation, hours of leaf wetness) showed negative associations with presence of WNV-positive mosquitoes. The non-linear variables (evapotranspiration, solar radiation, temperature) all showed that presence of WNV-positive mosquitoes was negatively associated with lower values of the variables, increasing positively as values increased, up to higher values where the positive relationship plateaued (S7 Fig). This relationship was observed for average minimum temperature over the previous 0–3 weeks in the final multivariable model, where minimum temperatures below approximately 13 degrees Celsius were negatively associated with presence and prevalence of WNV-positive mosquitoes, temperatures between approximately 13 and 17 degrees Celsius were increasingly positively associated up to a constant positive association at higher values (Fig 3). Minimum temperature over the previous 0–3 weeks was the variable that consistently produced the univariable model with the lowest WAIC score. The next variable added to the final model during the model building steps was solar radiation over the previous 0–1 weeks (which was non-linearly associated with prevalence of WNV-positive mosquitoes with higher values associated with reduced prevalence (Fig 3D)), however whilst the WAIC was lowest for this variable, the difference compared to other non-linear meteorological variables was minimal.

**Fig 4:**
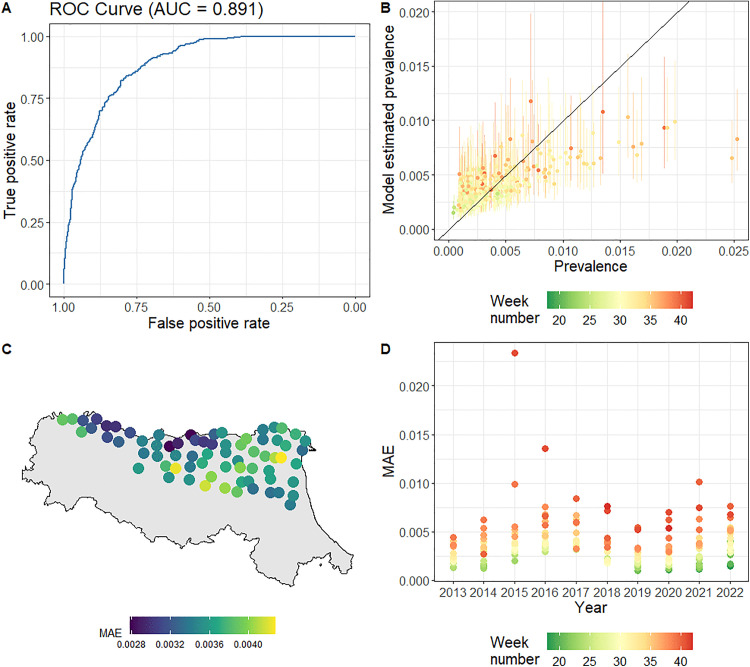
Assessment of model fit of the final multivariable model. (A) Receiver Operating Characteristic (ROC) curve with the calculated area under the curve (AUC) for the presence/absence part of the hurdle model, (B) median estimated prevalence and corresponding 95% Credible intervals compared to the observed prevalence, coloured by the week number, (C) mean absolute error (MAE) in the prevalence estimates per trap in Emilia-Romagna (grey background) averaged over the timeseries, and (D) MAE in the prevalence estimates per week and year coloured by the week number. The shapefile used in panel C was obtained from GADM (https://gadm.org/download_country.html and https://gadm.org/license.html).

The WAIC of the final multivariable model was -1083, compared to -768 for the baseline model. The area under the ROC curve (AUC) of the final model (Fig 4A) was 0.89, which showed that the presence/absence component of our hurdle model was a good binary classifier. The AUC of the baseline model was 0.83. We found that the final model estimated prevalence reproduced the observed prevalence (estimated with the *PooledInfRate* R package) well, especially at lower values and at the start and middle of the transmission season, and tended to underestimate the prevalence during the end of the transmission season (Fig 4B). The mean absolute error (MAE) averaged over both space and time was 3.53*10^-3, compared to 3.69*10^-3 for the baseline model. The MAE per trap location averaged over time of the final model ranged from 2.78*10^-3 to 4.27*10^-3 (Fig 4C), compared to 3.13*10^-3 to 4.56*10^-3 for the baseline model. The MAE per week number averaged over the traps and years of the final model ranged from 1.30*10^3 in the 18^th^ week to 8.91*10^3 in the 41^st^ week (Fig 4D), compared to 2.95*10^-3 in the 42^nd^ week to 3.92*10^-3 in the 39^th^ week for the baseline model.

**Fig 5:**
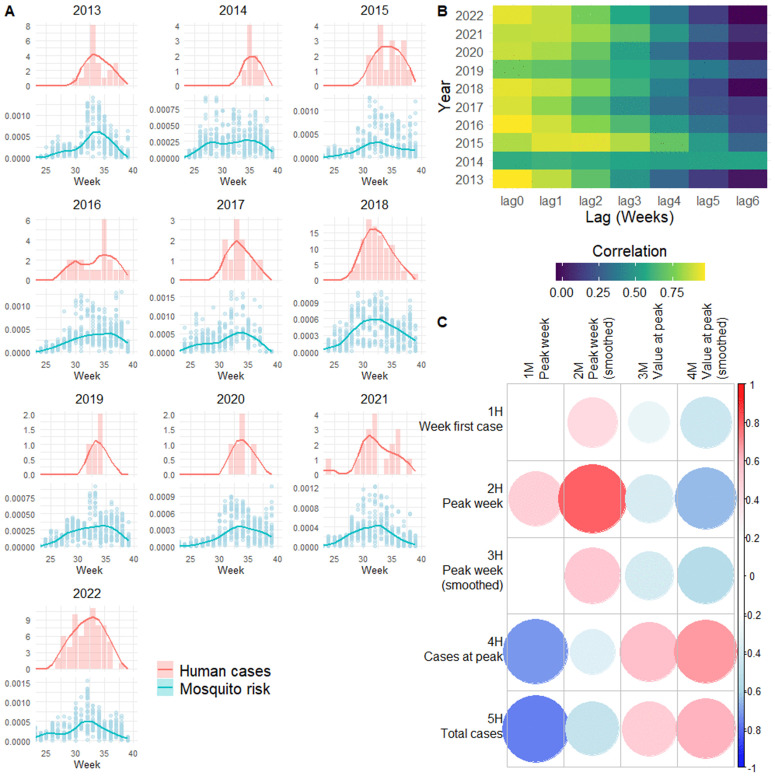
Comparison of the reported number of human West Nile neuroinvasive disease cases in Emilia-Romagna and the model-estimated risk of WNV-positive mosquitoes. Reported cases (pink bars) with model output (blue points) smoothed lines for each year (A). Correlation values at different lags from the cross-correlation analysis on the yearly timeseries of human cases and model output (B). Correlation values for the annual calculated human (1 to 5H) and model output mosquito (1 to 4M) risk metrics.

The number of human WNND cases in the peak week and the total number of cases at the end of each season negatively correlated with the model-estimated timing of WNV in mosquitoes (Fig 5C), meaning that earlier circulation in mosquitoes is associated with larger infection rates in humans. In the region, the largest number of reported WNND cases were reported in 2018 and 2022 (totalling 106 and 73 cases, respectively, compared to 24 cases in 2013 which was the next highest year) (Fig 5A). In these years, the estimated peak week of mosquito WNV risk was week 31, which is earlier in the season compared to almost all other years (median peak week = 33). The timeseries of reported WNND cases and the model-estimated mosquito WNV risk were strongly positively correlated over 0- to 2 week lags (Fig 5B). This is consistent with the positive correlations between 1-3H and 1-2M (timing of the human reported cases and model-estimated mosquito WNV risk each season) and 4-5H and 3-4M (number of reported WNND cases and the magnitude of model-estimated mosquito WNV risk) (Fig 5C).

The spatiotemporal fields in our models for presence of WNV-positive mosquitoes show the highest values in the north of Emilia-Romagna (S8–S10 Figs). The autocorrelation parameter (S11 Fig) for the spatiotemporal fields suggests that presence of WNV-positive mosquitoes is highly correlated across Emilia-Romagna between sequential years.

## Discussion

We present the results of a spatiotemporal modelling study which assessed the associations between presence and prevalence of WNV-positive mosquitoes, and meteorological, environmental, and avian WNV presence in Emilia-Romagna, Italy, over ten years of entomological field surveillance from 2013 to 2022. We tested the performance of Bayesian spatiotemporal mixed-effects regression models assessing the linear and non-linear associations of individual variables in univariable analyses, and of variables considered in combination in multivariable models. The temporal span and high spatial resolution of the data in the analysis provides significant insight into the relationships between the environment and mosquito WNV infection patterns in the region. We also compared the temporal patterns of reported human WNND cases and mosquito WNV infection metrics.

Presence of WNV in local birds was strongly positively associated with presence and prevalence of WNV-positive mosquitoes, which is consistent with the avian-mosquito WNV transmission cycle [46]. The avian WNV data was available at the municipality level (centroids of the municipality where hunting routes occurred) and was matched to traps within 20km, which is a reasonable distance for territorial species.

The proportion of artificial land use around mosquito traps was negatively associated with presence of WNV-positive mosquitoes in the univariable analysis, and the proportion of agricultural land use was consistently positively associated in all models. This relationship was consistent at finer scale resolution, where discontinuous urban fabric, industrial and commercial units, and road and rail networks were also negatively associated with presence of WNV-positive mosquitoes, and arable land was positively associated. Artificial and agricultural land use were collinear, and likely capture the same effect of unsuitable versus suitable habitat for *Culex* mosquitoes. Our work adds to the growing body of evidence that land use is an important factor for arbovirus risk [20], for example, Lu *et al*., 2024 showed that WNV spread in Europe was associated with the intensity of agricultural activities [7], Calzolari *et al*., 2010 reported a positive association between rural areas and WNV positive mosquito pools in Emilia-Romagna [10], and Marcantonio *et al*., 2015 showed that the presence of irrigated croplands was a significant predictor of human WNV incidence [25]. The majority of land use in Emilia-Romagna is characterised as agricultural under CLC level I categories, which includes the following CLC level III categories: non-irrigated arable land (74% of land around the traps), complex cultivation patterns (8.5%), rice fields (1.3%) and orchards, vineyards, and pastures (all < 1%). In the study area, across land classified as non-irrigated arable land by CLC, dense networks of channels are present that constitute a suitable environment for *Culex pipiens*. In future work it would be interesting to explore the associations between WNV risk and land use at finer resolution (e.g., using CLC level IV classifications or specific crops present in the different seasons) and better identify the specific agricultural practices associated with increased WNV circulation as well as potential mitigation strategies, as proper landscape managing. It would also be useful to conduct an entomological sensitivity trial on *Culex* presence in the region outside of the trap locations.

Minimum temperature was the best predictor of presence and prevalence of WNV-positive mosquitoes in our models (S3 Table) which suggests that this variable may act as a threshold condition in WNV infection dynamics, with minimum temperatures below around 13°C being associated with reduced prevalence of WNV-positive mosquitoes. These results are consistent with laboratory studies demonstrating the thermal dependency of *Culex* mosquito traits and mosquito-virus interactions [14–16], for example Mordecai *et al*., 2019 showed that *Culex pipiens* WNV transmission was limited below 16.8°C (95% CrI 14.9, 17.8°C) [14], and modelling analyses which report a lower thermal limit of 14°C for WNV establishment in European *Culex* populations [47] and a 15–28°C summer temperature range for increased human WNF cases [20]. Interestingly, minimum temperature has risen faster than maximum temperature both globally and in Emilia-Romagna over the last decades [48].

In addition to minimum temperature, solar radiation was included non-linearly in the final model, with prevalence of WNV-positive mosquitoes decreasing at the highest radiation values. Causal relationships cannot be inferred from regression models, but it is likely that this variable is serving as a proxy for other meteorological conditions which may impact *Culex* biology and/or transmission potential. To the best of our knowledge, our work is the first to explore the non-linear relationships between prevalence of WNV-positive mosquitoes and meteorological conditions in Emilia-Romagna.

The spatiotemporal fields for presence of WNV-positive mosquitoes show the highest values in the north of Emilia-Romagna (S8–S10 Figs) where the Po river runs through the region. Phylogenetic work showed that WNV entered Italy in the east in approximately 2008, and spread from the Po Valley following the river [1], and has therefore been circulating in this region for a longer time period. The conformation of the Po plain shows an inclination from West to East toward the sea, and additionally and more pronounced, from the Apennine mountains to the Po river, and therefore higher values of the spatial field close to the Po could be capturing the permissive ecological conditions and suitability of lowland areas for WNV circulation. This explanation is supported by our univariable analysis where we explicitly incorporated elevation and observed a negative association with mosquito WNV presence. The autocorrelation parameter (S11 Fig) for the spatiotemporal fields in our models suggests that presence of WNV-positive mosquitoes is highly correlated across Emilia-Romagna between sequential years, which suggests that areas with WNV-positive mosquitoes in the previous season and their surroundings should be targeted for surveillance the following season, and that once WNV is detected in an area it is likely to remain there, potentially due to successful mosquito overwintering [26,49–51] or persistence in wild birds [51–53].

The estimated prevalence from our final spatiotemporal model accurately reproduced prevalence calculated from entomological data, with higher error at the end of the transmission seasons when the pool size of trapped mosquitoes was smaller (S12 and S13 Figs). The models in our sensitivity analysis, which did not include spatiotemporal fields, less accurately reproduced the data, thus demonstrating the utility of the spatiotemporal methodology implemented in this study (Supplementary Material). The number of reported human WNND cases in the region each season was negatively correlated with the estimated timing of WNV risk in mosquitoes, meaning that entomological surveillance is particularly useful at detecting the onset of the transmission season, which in turn determines the magnitude of the number of spillover events into the human population (with earlier circulation in mosquitoes being associated with larger infection rates in humans). These results demonstrate a clear link between mosquito WNV prevalence and human risk, which supports the value of entomological surveillance and the future use of the modelling tools developed in this study as an early-warning system for human health risks.

The major strength of this work is the analysis of longitudinal entomological surveillance data at fine-scale spatial and temporal resolution, where mosquitoes were sampled at the same geolocations over a ten-year period, and the fitting of epidemiological models to this at-scale field data. The assessment of non-linear relationships between meteorological variables and the presence of WNV-positive mosquitoes, as opposed to more commonly studied outcome variables such as mosquito abundance, is a further strength. The main limitation of this analysis is that causal relationships cannot be inferred from regression models, and therefore mechanistic models which leverage physiological responses of vectors to their environment to explain disease transmission, are needed to further characterise the relationships highlighted in this study. The spatiotemporal scale of the predictors in our empirical models provides robust information about local-level risk factors, such as land use and non-linear meteorological drivers, which can be utilised to complement mechanistic WNV transmission models in the future. Future work could also explore longer time-lags in the meteorological variables, and include measures of extremes of meteorological variables such as drought indices. The avian WNV data used in this analysis was available at the municipality level and we assumed that the WNV infection patterns in the sampled birds were representative of the whole avian population; it would therefore be interesting to compare our results to work utilising finer resolution, more representative, data if they were available.

Our findings provide evidence of the combined importance of avian WNV presence, meteorological conditions, and land use in influencing mosquito WNV infection dynamics at the local level in Emilia-Romagna. At the region level, averaged mosquito WNV infection dynamics were strongly correlated with the seasonality of reported cases of human WNND. The methods developed in this work lay the foundations for the development of a real-time projection tool that may be useful to forecast WNV risk in the region under warming temperatures and land use changes to inform targeted and optimised control policy in future.

## Supporting information

S1 Text Supplementary methods. (DOCX)

S1 Table Assessment of spatiotemporal models with different fields. Fields were modelled as iid or ar1 (independent per year or autoregressively related across the years). The WAIC scores are a measure of prediction error.
(DOCX)

S2 Table WAIC per model building step in the additional analysis using Vector Index as the outcome variable in the models. (DOCX)

S3 Table WAIC per model building step in the main analysis using prevalence as the outcome variable in the models. (DOCX)

S1 Fig West Nile virus presence in tested birds in Emilia-Romagna each year. Bars show the counts of collected birds per species: *Corvus cornix* (hooded crow), *Garrulus glandarius* (Eurasian jay), and *Pica pica* (magpie). Blue bars show counts of West Nile virus positive birds, and orange show West Nile virus negative birds.
(PNG)

S2 Fig Number of weeks of West Nile virus (WNV) presence in the avian population. The points represent the mosquito trap locations and are coloured to show the number of weeks of avian WNV presence at province centroids within a 20 km radius to each trap. Avian WNV presence was assumed from the first week in which WNV was detected to the end of the transmission season each year in the avian surveillance data (A), or using only the recorded presence from the surveillance data (B). The shapefile used to generate the base layer maps was obtained from GADM (https://gadm.org/download_country.html and https://gadm.org/license.html).
(PNG)

S3 Fig Meteorological variables in Emilia-Romagna over 2013–2023. The variables are shown for the week of trapping and averaged over the trap locations.
(PDF)

S4 Fig Elevation above sea level and the proportion of each CLC level I land cover category in a 3km buffer around each trap. Key: CLC1 artificial surfaces, CLC2 agricultural areas, CLC3 forest and seminatural areas, CLC4 wetlands and CLC5 water bodies. The shapefile used to generate the base layer maps was obtained from GADM (https://gadm.org/download_country.html and https://gadm.org/license.html).
(PNG)

S5 Fig Land use composition. The proportion of land within a 3km buffer around each trap, categorised by each of the CLC level I land use categories.
(TIFF)

S6 Fig Mosquito West Nile virus prevalence (infection rate) estimates. (A) Occurrence, 1, and absence, 0, of prevalence greater than zero. (B) Value of prevalence at occurrence. (C) Log transformation of the prevalence at occurrence.
(PNG)

S7 Fig Non-linear relationships between meteorological variables and (A) presence of WNV-positive mosquitoes and (B) prevalence of WNV-positive mosquitoes in the univariable models. Random walk coefficients for evapotranspiration, radiation, average temperature, maximum temperature, minimum temperature, absolute maximum temperature, and absolute minimum temperature over different time lags (lagged by 0, 1, 2, 3 weeks or averaged over the previous 0–1, 0–2, or 0–3 weeks) in the univariable models.
(TIFF)

S8 FigMean values of the spatiotemporal random fields on presence of WNV-positive mosquitoes, $\;\mu_{z,i,a}$ (A) and transformed prevalence of WNV-positive mosquitoes, $\mu_{y,i,a}$ (B) for each year 2013–2022 read top left to bottom right, in the baseline model.The shapefile used to generate the administrative border was obtained from GADM (https://gadm.org/download_country.html and https://gadm.org/license.html).(PNG)

S9 FigLower credible interval values of the spatiotemporal random fields on presence of WNV-positive mosquitoes, $\mu_{z,i,a}$ (A) and transformed prevalence of WNV-positive mosquitoes, $\mu_{y,i,a}$ (B) for each year 2013–2022 read top left to bottom right, in the baseline model.Lower credible interval is the 2.5% quantile. The shapefile used to generate the administrative border was obtained from GADM (https://gadm.org/download_country.html and https://gadm.org/license.html).(PNG)

S10 FigUpper credible interval values of the spatiotemporal random fields on presence of WNV-positive mosquitoes, $\mu_{z,i,a}$ (A) and transformed prevalence of WNV-positive mosquitoes, $\mu_{y,i,a}$ (B) for each year 2013–2022 read top left to bottom right, in the baseline model.Upper credible interval is the 97.5% quantile. The shapefile used to generate the administrative border was obtained from GADM (https://gadm.org/download_country.html and https://gadm.org/license.html).(PNG)

S11 FigPosterior marginal distribution of the parameters in the baseline spatiotemporal model.(A) GroupRho for $\mu_{z}$ specifies the relationship between $\mu_{z}\;$ fields in successive years, (B) range for $\mu_{z}$ and (C) $\mu_{y}$ is given in kilometres, (D) standard deviation for $\mu_{z}$ and (E) $\mu_{y}$, and (F) precision for the Gamma observations which is the inverse of the variance.(PNG)

S12 Fig Mean and 95% quantile of the pool size per week number averaged over the traps and years. (PNG)

S13 Fig Summary statistics of the calculated prevalence of WNV-positive mosquitoes. Proportion of traps where the calculated prevalence was greater than zero, per week and year (A) and the calculated prevalence when greater than zero per week number, grouped over the years, shown with data points in blue and boxplot summaries in grey (B).
(PNG)

S14 Fig Triangulated mesh. Emilia-Romagna area is shown in light blue, coordinates of the trap locations represented as black points, and the random field boundary in dark blue. The shapefile used to generate the administrative border was obtained from GADM (https://gadm.org/download_country.html and https://gadm.org/license.html).
(TIFF)

S15 Fig Regression coefficients for each percentile of the meteorological variables included as random effect categorical variables in univariable spatiotemporal models, associated with prevalence of WNV-positive mosquitoes. The meteorological variables were calculated at time lag = 0 (in the week of mosquito trapping).
(PNG)

S16 Fig Regression coefficients for each percentile of the meteorological variables included as random effect categorical variables in univariable spatiotemporal models, associated with the presence of WNV-positive mosquitoes. The meteorological variables were calculated at time lag = 0 (in the week of mosquito trapping).
(PNG)
