# Supplementary material for: Meteorological and environmental drivers of West Nile virus prevalence in Culex pipiens mosquitoes in Emilia-Romagna, Italy in 2013 to 2022
Source: PLoS Pathog. 2025 Dec 5;21(12):e1013753. doi: 10.1371/journal.ppat.1013753 (PMC12680267; doi:10.1371/journal.ppat.1013753)
Supplement: S1 Text — (DOCX) [file ppat.1013753.s001.docx]

**Supplementary Methods**

**Contents**

1. Assessing spatial autocorrelation in the entomological surveillance data
2. Baseline spatiotemporal SPDE regression model
3. Field choice in the baseline spatiotemporal model
4. Testing linear and non-linear relationships
5. Non-linear variables as random walks
6. Vector Index as an alternative response variable
7. Sensitivity analysis for the avian WNV presence variable
8. Sensitivity analysis developing the models without the spatiotemporal fields

**References**

1. **Assessing spatial autocorrelation in the entomological surveillance data**

For each trap the following metrics were calculated from the entomological data: (1) mean average infection rate averaged across weeks and years, (2) proportion of weeks across all sampled weeks and years where the infection rate was greater than zero i.e. estimated presence of WNV, (3) annual mean average infection rate averaged across weeks, and (4) annual proportion of weeks across all sampled weeks where the infection rate was greater than zero.

We assigned each trap its nearest six neighbours, based on distance between the coordinates, and computed the Moran I statistic for the trap-level metrics using the *spdep* R package (1); which indicates presence of spatial autocorrelation when the values are positive with significant p-values (<0.05). For metric 1 and 2 there was significant spatial autocorrelation (Moran I: 0.136 (p-value = 0.00749) and 0.173 (p-value = 0.00102)), respectively. For metric 3, there was strong spatial autocorrelation in 2016, 2017, 2018, and 2021, and for metric 4 there was strong spatial autocorrelation in 2013, 2016, 2017, and 2021.

1. **Baseline spatiotemporal SPDE regression model**

Presence and prevalence of WNV-positive mosquitoes was assumed to be continuous across space (a Gaussian field (GF)) determined by intra- and inter-annual temporal relationships and spatial correlation. We developed spatiotemporal models in R-INLA using the stochastic partial differential equations (SPDE) approach (2–4), which represents GFs as discrete processes (Gaussian Markov Random Fields (GMRFs), herein called fields).

The fields ($\xi(s)$) are solutions to the SPDEs which specify spatial correlation between any two locations$s_{i}$ and $s_{j}$ and are characterised by the Matérn covariance function in Equation S1, where $\left\| s_{i}-s_{j} \right\|$ is the Euclidian distance between $s_{i}$ and $s_{j}$, $\sigma_{\xi}^{2}$ is the variance, $K_{v}$ is a modified Bessel function with smoothness parameter $v$, and $\kappa$ is a scaling parameter determining the range $\delta$, at which $s_{i}$ and $s_{j}$ are spatially independent. The range can be calculated empirically as $\delta= \sqrt{\frac{8v}{\kappa}}$ (2).

$$Cov\left( {\xi(s}_{i} \right), {\xi(s}_{j}))=\frac{\sigma_{\xi}^{2}}{\Gamma\left( v \right)2^{v-1}}\left( \kappa\left\| s_{i}-s_{j} \right\| \right)^{v}K_{v}\left( \kappa\left\| s_{i}-s_{j} \right\| \right)$$

Equation S1.

We used penalised complexity priors on the Matérn function hyperparameters (5), where the prior distribution on $\delta$ is defined as a probability (P) statement of P(d, 0.05), which specifies that there is 5% probability that the spatial range is less than d (which is the approximate minimum Euclidean distance in degrees between any two of the traps). The prior distribution on $v$ was defined flexibly using P(d, 0.5) which states there is a 50% probability that the standard deviation of the spatial range is >d.

The fields were built on triangulated meshes representing the trap locations (Figure S14). We calculated the difference between the minimum and maximum distance between any two trap locations, herein called π. We specified the minimum angle of the triangles as 25 degrees (to avoid building many small triangles around the coordinates), the offset of the inner domain as π/50, the offset of the outer domain as π/5, and the maximum edge length of the triangles for the inner and outer domain as π/10 and π respectively. We set a boundary of the fields on the mesh to the coastal border of the region using a GADM shapefile (6), and the cut-off value as π/100 to build small triangles at the boundary edge (to reduce boundary effects).

1. **Field choice in the baseline spatiotemporal model**

To determine the best spatiotemporal model, we tested several combinations of fields capturing the spatial correlation and inter-annual temporal heterogeneities of presence and prevalence of WNV-positive mosquitoes. We tested including different spatial fields per year, $a$; (1) separately for $z_{i}$ ($\mu_{z,i,a}$) and for $y_{i}$ ($\mu_{y,i,a}$), (2) shared across $z_{i}$ and $y_{i}$ ($\omega_{i,a}$), and (3) combining separate fields for $z_{i}$ ($\mu_{z,i,a}$) and for $y_{i}$ ($\mu_{y,i,a}$) with a shared field across $z_{i}$ and $y_{i}$ ($\omega_{i,a}$), under the assumption that the fields per year are independent of each other (*iid*), or assuming an autoregressive model (*ar1)* where the fields per year are related to sequential years through an autoregressive function of order 1 (i.e. the field in year $a$ depends on the field in the previous year $a$-1). Table S1 shows the linear predictors of each tested model and the assessed Watanabe-Akaike Information Criterion (WAIC) scores of each model, which is a measure of predictive error (7).

Model 6 (which had separate fields for $z_{i}$ and $y_{i}$ and a shared field $\omega_{i,a}$, modelled using an *ar1* relationship) had the lowest WAIC (Table S1) however the parameter determining the scaling of the shared field $\omega_{i,a}$ compared to $\mu_{z,i,a}$ in model 6 was insignificant (the credible interval crossed zero). Model 4 had the next lowest WAIC, however when we tested whether the *ar1* terms were significant, we saw that in the $\mu_{z,i,a}$ field it was significant but in the $\mu_{y,i,a}$ field it was not. We then fit model 7, which was a repeat of model 4 except $\mu_{y,i,a}$ was modelled using an *iid* model, whilst $\mu_{z,i,a}$ was modelled using an *ar1* model as before. Model 7 was chosen as the baseline spatiotemporal model for further model development, its parameters and the spatial visualisation of the fields are shown in Figures S8-S11. The autoregressive parameters specifying the temporal correlation of the $\mu_{z,i,a}$ field between year $a$ and $a$+1, was 0.95 (95% Credible Interval, 0.86, 0.99), which suggests that the spatial pattern of presence of WNV-positive mosquitoes in sequential years is highly correlated. The large range of the fields (218km (95% CrI, 124, 399km) and 46km (95% CrI, 20, 111km) for $\mu_{z,i,a}$ and $\mu_{y,i,a}$ respectively) show that WNV circulation in mosquito populations, particularly presence, is highly spatially correlated across the region.

1. **Testing linear and non-linear relationships**

To assess whether meteorological variables should be included in the regression models as linear or non-linear effects, we performed an initial analysis where each variable was categorised according to its 10^th^ to 90^th^ percentiles and added to the baseline spatiotemporal model as a random effect categorical variable associated with either the presence and prevalence of WNV-positive mosquitoes. The linearity of the regression coefficient pattern over the percentiles was assessed (shown for variables at time lag 0 in Figures S15 and S16). Solar radiation, evapotranspiration, and temperature variables were non-linearly associated.

1. **Non-linear variables as random walks**

The non-linearly associated variables (temperature, evapotranspiration, and solar radiation variables, as categorised using the methods described in section 3) were grouped into 30 bins and included in the regression models as random effects using random walks of order 2. The prior on the random effect precision was set using penalised complexity priors of P(1, 0.1) which was deemed suitable to give flexible random walks in an initial sensitivity analysis where the non-linear variables were added to the baseline model separately to generate univariable models, and the resulting random walk coefficients were assessed. The following priors were tested in the sensitivity analysis: P(0.001, 0.01), P(0.01, 0.01), P(0.1, 0.01), P(0.1, 0.1), P(1, 0.01), P(1, 0.1), and P(1, 0.5).

1. **Vector Index as an alternative response variable**

Vector Index (VI) is the prevalence of WNV-positive mosquitoes multiplied by the number of mosquitoes tested * 100, per trap and time point. We estimated VI and performed a sensitivity analysis fitting the spatiotemporal hurdle models to the VI as the response variable. The WAIC of the final multivariable model fitted to the VI was 5584.97, compared to 5875.47 for the baseline model fitted to the VI. The area under the ROC curve (AUC) of the final model was 0.891, compared to 0.826 for the baseline model. The mean absolute error (MAE) averaged over both space and time was 48.13, compared to 49.72 for the baseline model.

The model building steps are shown in Table S2; we observed that the same variables were chosen in the final model with VI as the response variable compared to prevalence of WNV-positive mosquitoes. For both the baseline and final models, the models fitted to VI had worse WAIC scores than the models fitted to prevalence of WNV-positive mosquitoes (Tables S2 and S3).

1. **Sensitivity analysis for the avian WNV presence variable**

Since avian WNV surveillance may have been insensitive to the detection of WNV-positive birds in particular weeks, in the main analysis we assumed that WNV was continuously present in the avian population from the first week in which WNV was detected to the end of the transmission season each year. To test this assumption, we compared univariable models with different avian WNV presence variables: either (A) the assumption done in the main analysis, as shown in Figure S2A, and (B) using only recorded presence. The WAIC scores for the univariable models were -812.382 and -804.364 for A and B, respectively. The regression coefficients for the association with presence of WNV-positive mosquito pools were 0.814 (95% CrI: 0.553, 1.074), and 1.089 (0.777, 1.400), for A and B, respectively. The regression coefficients for the association with prevalence of WNV-positive mosquito pools was 0.315 (95% CrI: 0.143, 0.488), and 0.0834 (-0.104, 0.271), for A and B, respectively.

1. **Sensitivity analysis – models with and without spatiotemporal** **fields**

To demonstrate the utility of using the INLA models presented in this study, and specifically the effect of accounting for spatiotemporal autocorrelation (through the spatiotemporal fields) on the model predictive performance, we ran the analysis building the models without the fields (effectively simple regression models with only the meteorological, land use, and avian variables). The variables chosen in the model building steps were: average minimum temperature 0-3, avian WNV presence, average solar radiation 0-1, CLC2 land use, and average relative humidity 0-3. The first four variables were the same as those chosen in the spatiotemporal models in the main analysis, and only the final variable (relative humidity) was an addition compared to the spatiotemporal models. This demonstrates that the chosen meteorological, land use, and avian variables in the spatial model are not contingent on the spatiotemporal component. It is important to note that the model fit without the spatiotemporal fields was worse than with the spatiotemporal model across all indicators, including: the marginal log likelihood (84.2 compared to 214.61), Area Under the Curve (AUC = 0.79 compared to 0.89), Watanabe-Akaike Information Criterion (WAIC = -740.42 compared to -1083) and mean absolute error of the prevalence estimates averaged over both space and time (MAE = 4.35*10^-3 compared to 3.53*10^-3), and the MAE per trap location averaged over time (range 3.46*10^-3 to 5.60*10^-3 compared to 2.78*10^-3 to 4.27*10^-3). We also calculated the Moran I statistic for the MAE averaged over space and time, and observed higher spatial autocorrelation in the error metric for the non-spatial model than for the final spatial model (Moran I = 0.512 compared to 0.395), which further emphases the need for spatial modelling.

**References**

1. Pebesma, E., Bivand R. Spatial Data Science: With Applications in R (1st ed.). Chapman and Hall/CRC.; 2023.

2. Lindgren F, Rue H, Lindström J. An explicit link between Gaussian fields and Gaussian Markov random fields: the stochastic partial differential equation approach. J R Stat Soc Series B Stat Methodol. 2011 Sep 1;73(4):423–98.

3. Bakka H, Rue H, Fuglstad GA, Riebler A, Bolin D, Illian J, et al. Spatial modeling with R-INLA: A review. Wiley Interdiscip Rev Comput Stat. 2018 Nov 1;10(6):e1443.

4. Blangiardo M, Cameletti M. Spatial and Spatio-temporal Bayesian Models with R-INLA. 2015.

5. Fuglstad GA, Simpson D, Lindgren F, Rue H. Constructing Priors that Penalize the Complexity of Gaussian Random Fields. https://doi.org/101080/0162145920171415907. 2018 Jan 2;114(525):445–52.

6. University of California Berkely. Global Administrative Areas (2020) [digital geospatial data]. 2020.

7. Watanabe SWATANAB S. A Widely Applicable Bayesian Information Criterion. Journal of Machine Learning Research. 2013;14(Mar):867–97.
