# Supplementary material for: Meteorological and environmental drivers of West Nile virus prevalence in Culex pipiens mosquitoes in Emilia-Romagna, Italy in 2013 to 2022
Source: PLoS Pathog. 2025 Dec 5;21(12):e1013753. doi: 10.1371/journal.ppat.1013753 (PMC12680267; doi:10.1371/journal.ppat.1013753)
Supplement: S1 Table — Fields were modelled as iid or ar1 (independent per year or autoregressively related across the years). The WAIC scores are a measure of prediction error. (DOCX) [file ppat.1013753.s002.docx]

**Table S1:** Assessment of spatiotemporal models with different fields. Fields were modelled as iid or ar1 (independent per year or autoregressively related across the years). The WAIC scores are a measure of prediction error.

| Model number | Formulae | Field model | WAIC |
| --- | --- | --- | --- |
| 1 | $z_{i, t} \sim\alpha_{z}+\mu_{z,i,a}$  $y_{i,t} \sim\alpha_{y}+ \mu_{y,i,a}$ | *iid* | -724.23 |
| 2 | $z_{i, t} \sim\alpha_{z}+ \omega_{i,a}$  $y_{i,t} \sim\alpha_{y}+ \omega_{i,a}$ | *iid* | -644.27 |
| 3 | $z_{i, t} \sim\alpha_{z}+ \mu_{z,i,a} + \omega_{i,a}$  $y_{i,t} \sim\alpha_{y}+ \mu_{y,i,a}+ \omega_{i,a}$ | *iid* | -725.11 |
| 4 | $z_{i, t} \sim\alpha_{z}+ \mu_{z,i,a}$  $y_{i,t} \sim\alpha_{y}+ \mu_{y,i,a}$ | *ar1* | -771.75 |
| 5 | $z_{i, t} \sim\alpha_{z}+\omega_{i,a}$  $y_{i,t} \sim\alpha_{y}+\omega_{i,a}$ | *ar1* | -692.06 |
| 6 | $z_{i, t} \sim\alpha_{z}+ \mu_{z,i,a} + \omega_{i,a}$  $y_{i,t} \sim\alpha_{y}+ \mu_{y,i,a}+ \omega_{i,a}$ | *ar1* | -772.01 |
| 7 | Model 4 | *ar1* for $\mu_{z,i,a}$, *iid* for $\mu_{y,i,a}$ | -768.01 |
