# Supplementary material for: Meteorological and environmental drivers of West Nile virus prevalence in Culex pipiens mosquitoes in Emilia-Romagna, Italy in 2013 to 2022
Source: PLoS Pathog. 2025 Dec 5;21(12):e1013753. doi: 10.1371/journal.ppat.1013753 (PMC12680267; doi:10.1371/journal.ppat.1013753)
Supplement: S3 Table — (DOCX) [file ppat.1013753.s004.docx]

**Table S3:** WAIC per model building step in the main analysis using prevalence as the outcome variable in the models.

|  | Model | WAIC |
| --- | --- | --- |
| Baseline | Spatiotemporal random fields | -767.99 |
| Best univariable | Baseline + average minimum temperature 03 | -1009.69 |
| Step 1 | Best univariable + average solar radiation 01 | -1051.26 |
| Step 2 | Step 1 + avian WNV presence | -1076.38 |
| Step 3 | Step 2 + artificial land use (CLC2) | -1083.50 |
