# Supplementary figures and images for: Meteorological and environmental drivers of West Nile virus prevalence in Culex pipiens mosquitoes in Emilia-Romagna, Italy in 2013 to 2022
Source: PLoS Pathog. 2025 Dec 5;21(12):e1013753. doi: 10.1371/journal.ppat.1013753 (PMC12680267; doi:10.1371/journal.ppat.1013753)

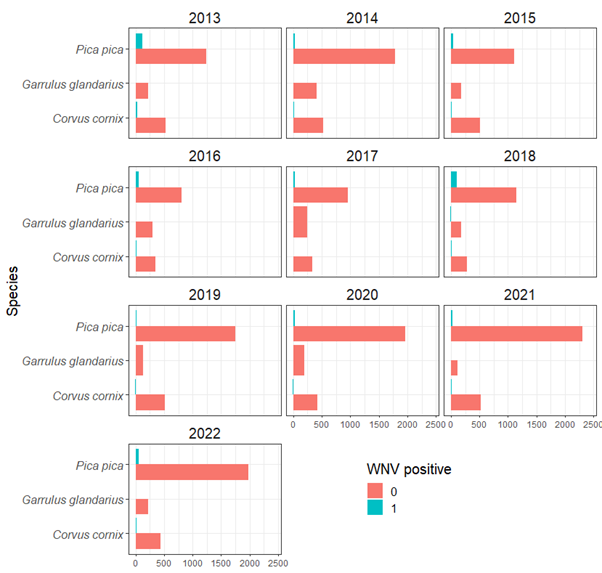

Supplement: S1 Fig — Bars show the counts of collected birds per species: Corvus cornix (hooded crow), Garrulus glandarius (Eurasian jay), and Pica pica (magpie). Blue bars show counts of West Nile virus positive birds, and orange show West Nile virus negative birds. (PNG) [file ppat.1013753.s005.png]

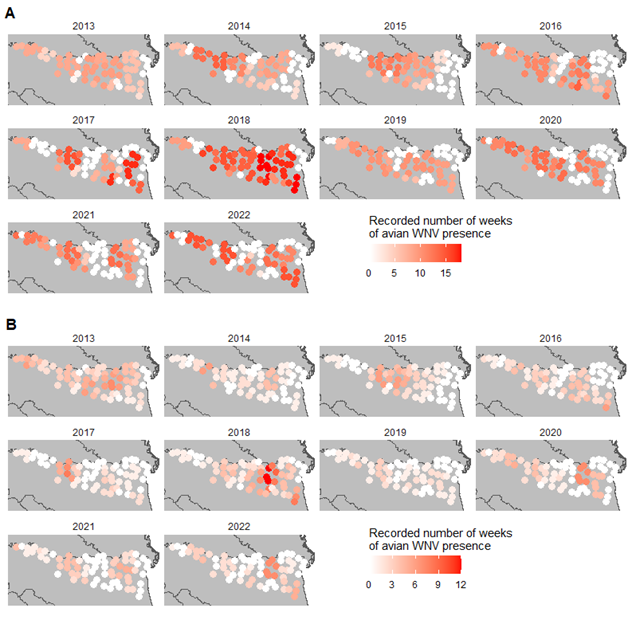

Supplement: S2 Fig — The points represent the mosquito trap locations and are coloured to show the number of weeks of avian WNV presence at province centroids within a 20 km radius to each trap. Avian WNV presence was assumed from the first week in which WNV was detected to the end of the transmission season each year in the avian surveillance data (A), or using only the recorded presence from the surveillance data (B). The shapefile used to generate the base layer maps was obtained from GADM (https://gadm.org/download_country.html and https://gadm.org/license.html). (PNG) [file ppat.1013753.s006.png]

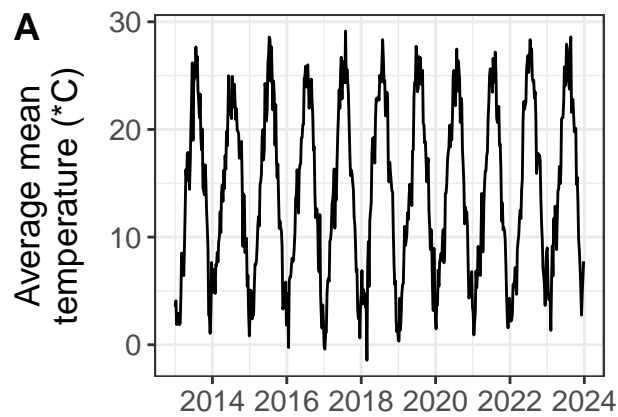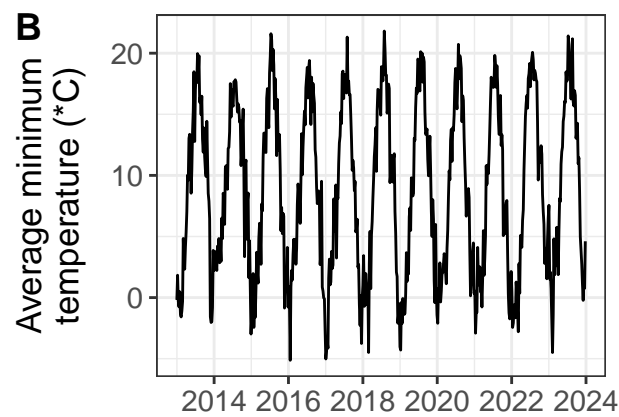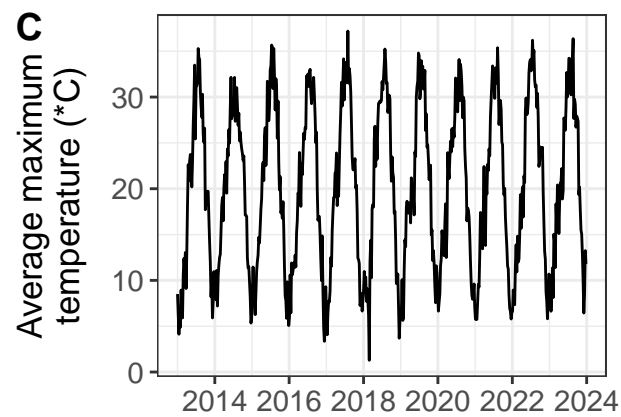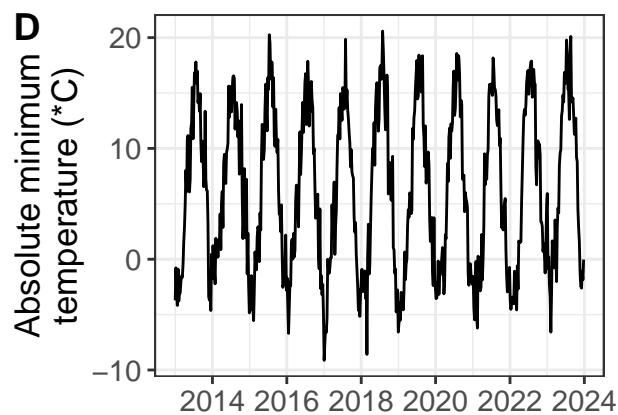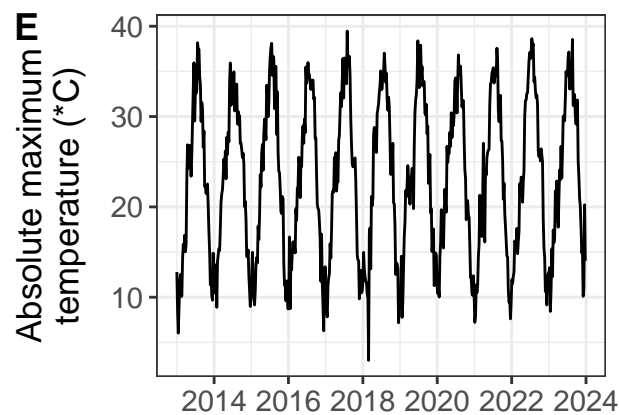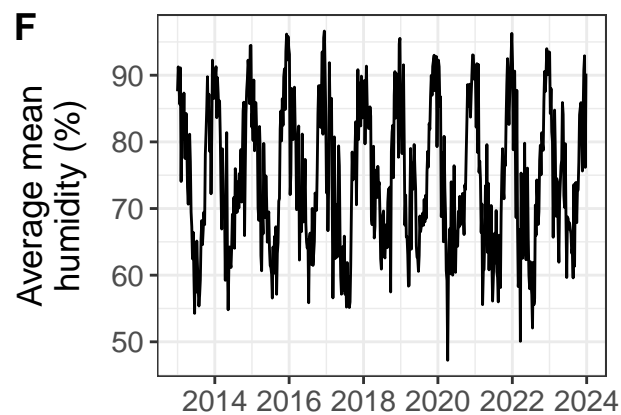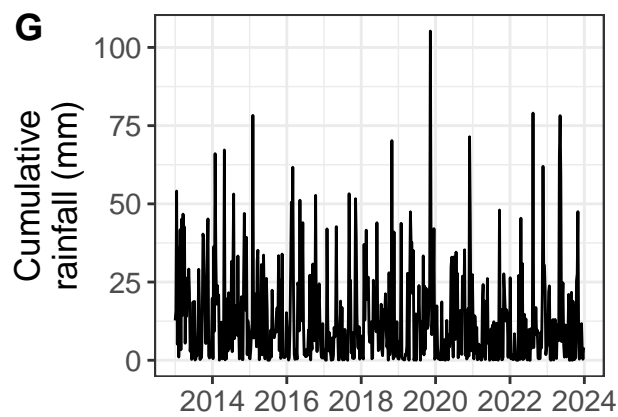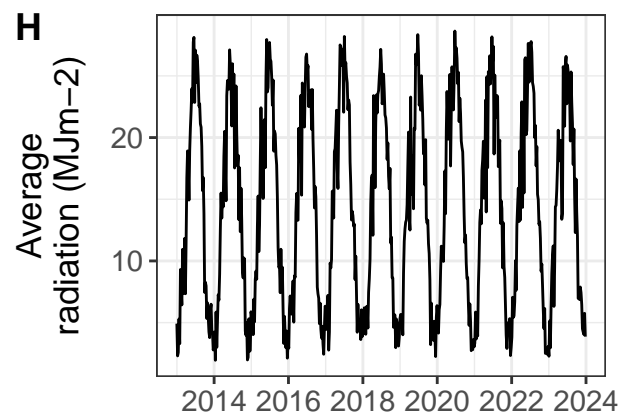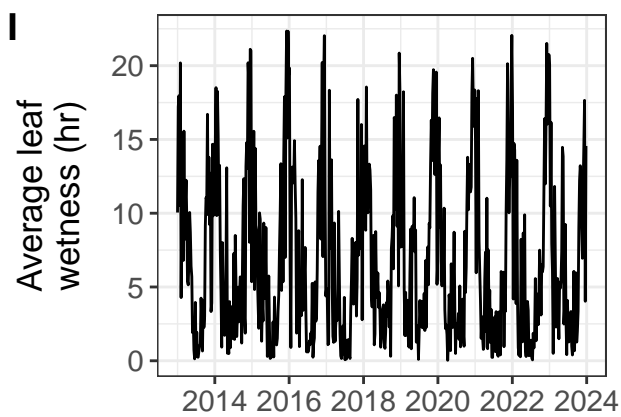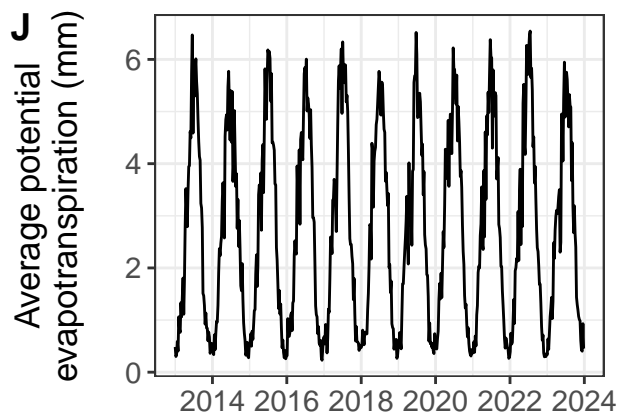

Supplement: S3 Fig — The variables are shown for the week of trapping and averaged over the trap locations. (PDF) [file ppat.1013753.s007.pdf]

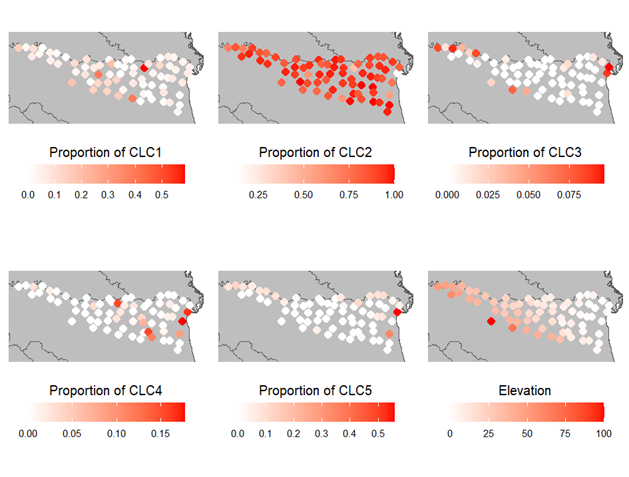

Supplement: S4 Fig — Key: CLC1 artificial surfaces, CLC2 agricultural areas, CLC3 forest and seminatural areas, CLC4 wetlands and CLC5 water bodies. The shapefile used to generate the base layer maps was obtained from GADM (https://gadm.org/download_country.html and https://gadm.org/license.html). (PNG) [file ppat.1013753.s008.png]

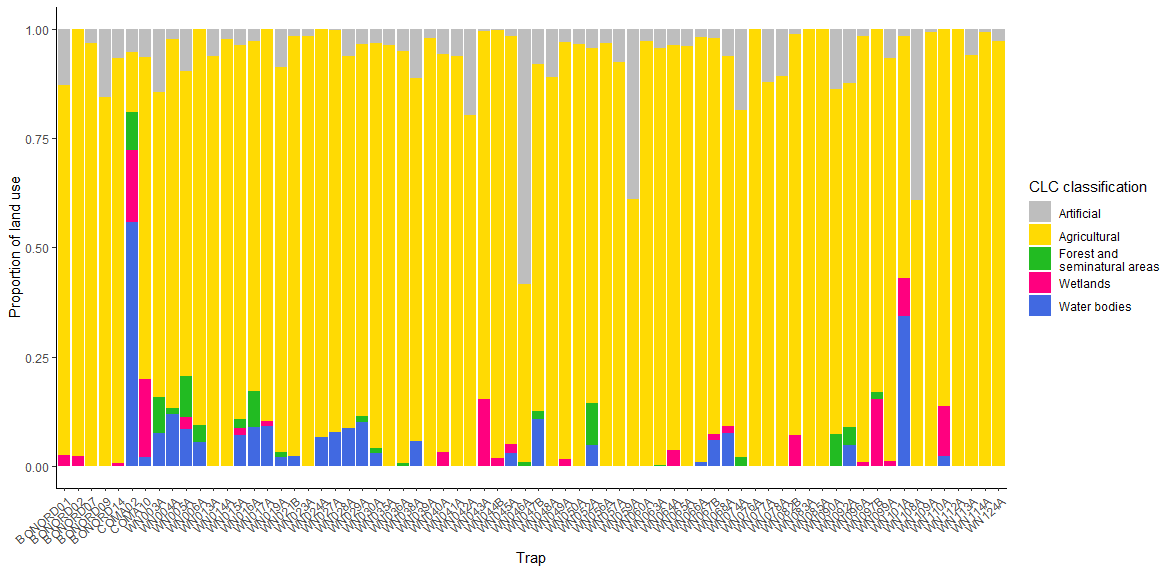

Supplement: S5 Fig — The proportion of land within a 3km buffer around each trap, categorised by each of the CLC level I land use categories. (TIFF) [file ppat.1013753.s009.tiff]

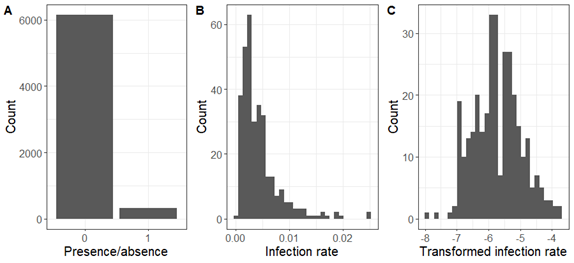

Supplement: S6 Fig — (A) Occurrence, 1, and absence, 0, of prevalence greater than zero. (B) Value of prevalence at occurrence. (C) Log transformation of the prevalence at occurrence. (PNG) [file ppat.1013753.s010.png]

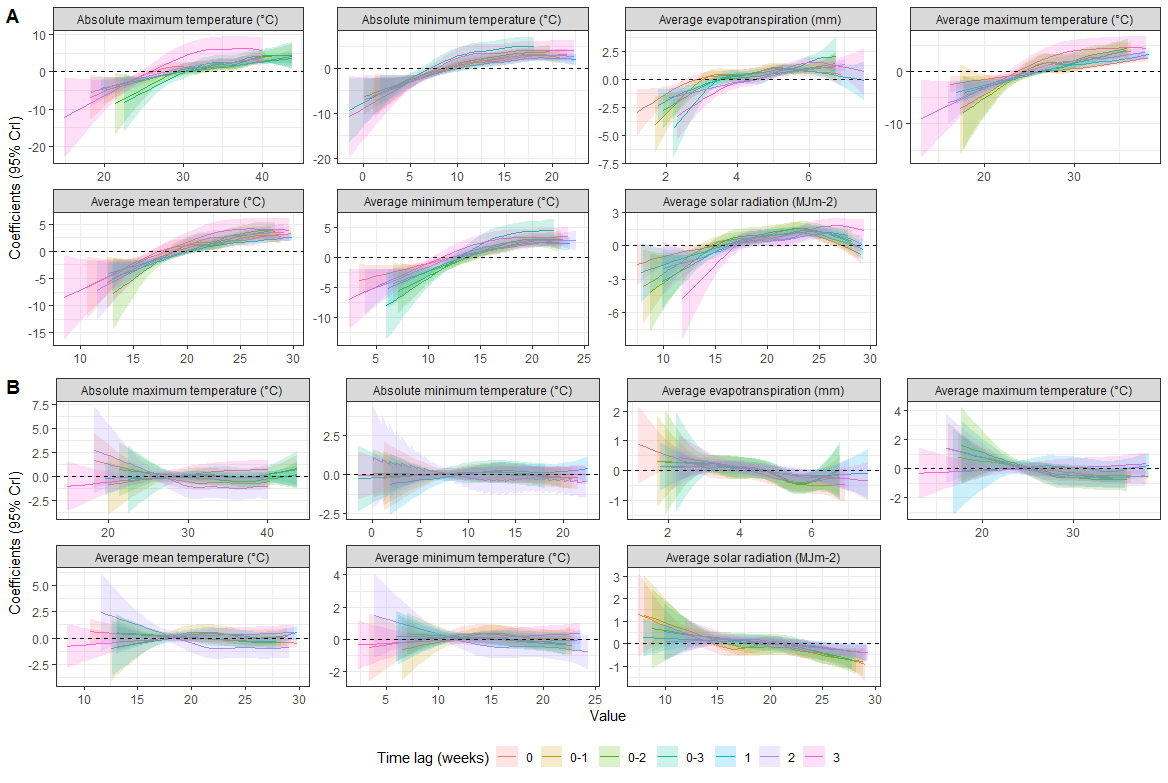

Supplement: S7 Fig — Random walk coefficients for evapotranspiration, radiation, average temperature, maximum temperature, minimum temperature, absolute maximum temperature, and absolute minimum temperature over different time lags (lagged by 0, 1, 2, 3 weeks or averaged over the previous 0–1, 0–2, or 0–3 weeks) in the univariable models. (TIFF) [file ppat.1013753.s011.tiff]

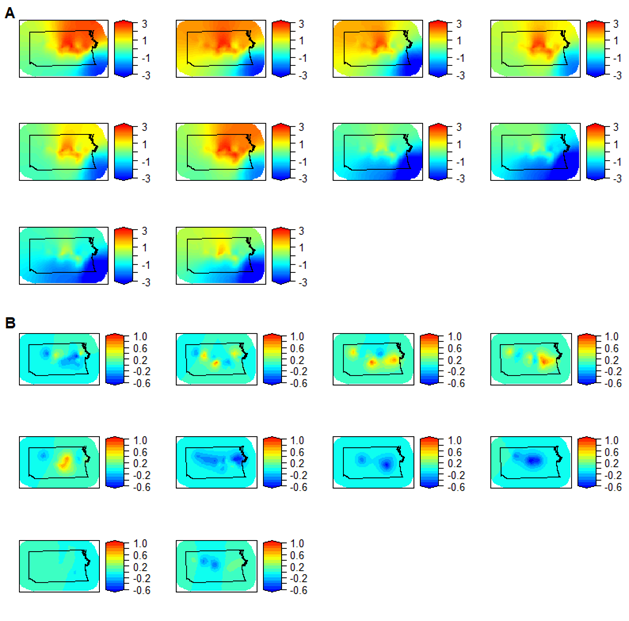

Supplement: S8 Fig — The shapefile used to generate the administrative border was obtained from GADM (https://gadm.org/download_country.html and https://gadm.org/license.html). (PNG) [file ppat.1013753.s012.png]

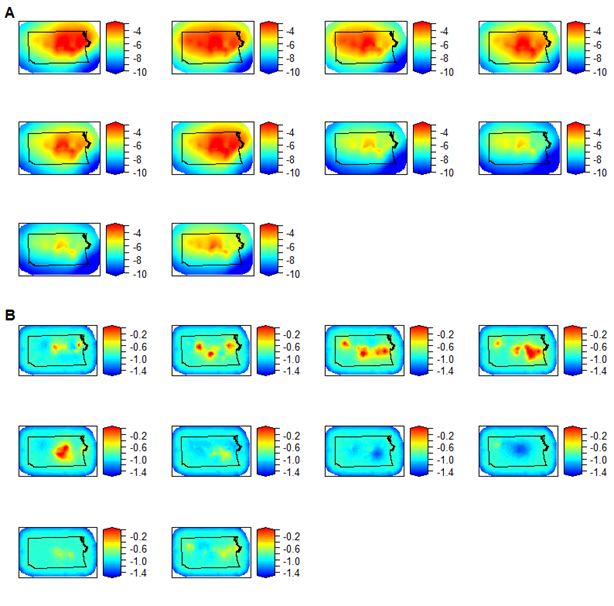

Supplement: S9 Fig — Lower credible interval is the 2.5% quantile. The shapefile used to generate the administrative border was obtained from GADM (https://gadm.org/download_country.html and https://gadm.org/license.html). (PNG) [file ppat.1013753.s013.png]

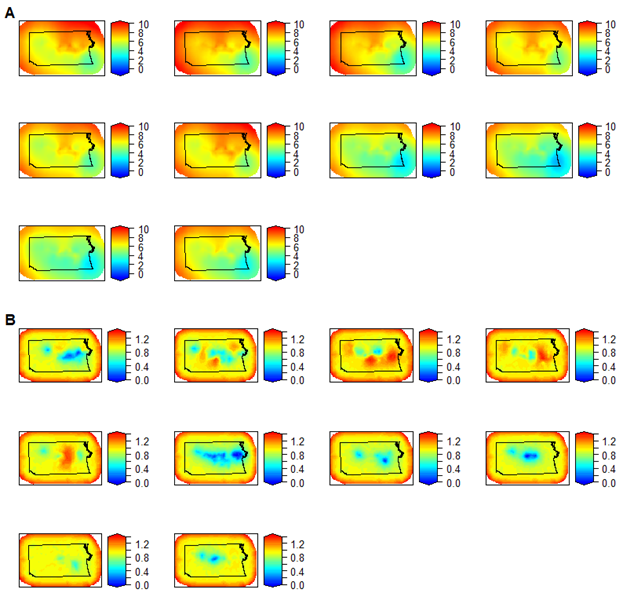

Supplement: S10 Fig — Upper credible interval is the 97.5% quantile. The shapefile used to generate the administrative border was obtained from GADM (https://gadm.org/download_country.html and https://gadm.org/license.html). (PNG) [file ppat.1013753.s014.png]

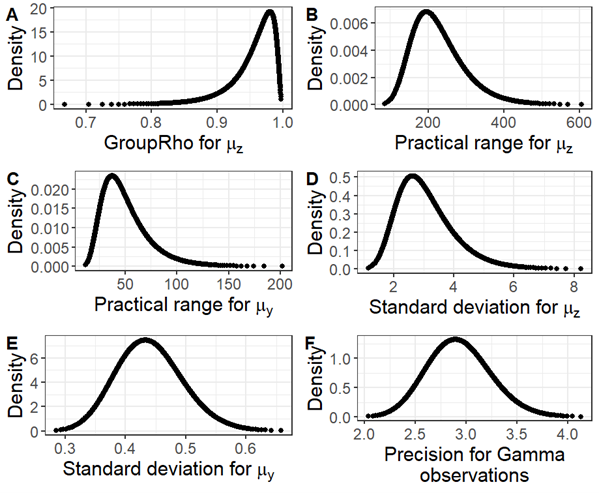

Supplement: S11 Fig — (A) GroupRho for μz specifies the relationship between μz fields in successive years, (B) range for μz and (C) μy is given in kilometres, (D) standard deviation for μz and (E) μy, and (F) precision for the Gamma observations which is the inverse of the variance. (PNG) [file ppat.1013753.s015.png]

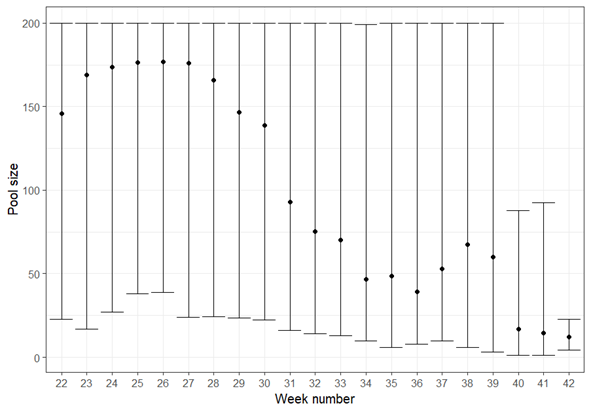

Supplement: S12 Fig — (PNG) [file ppat.1013753.s016.png]

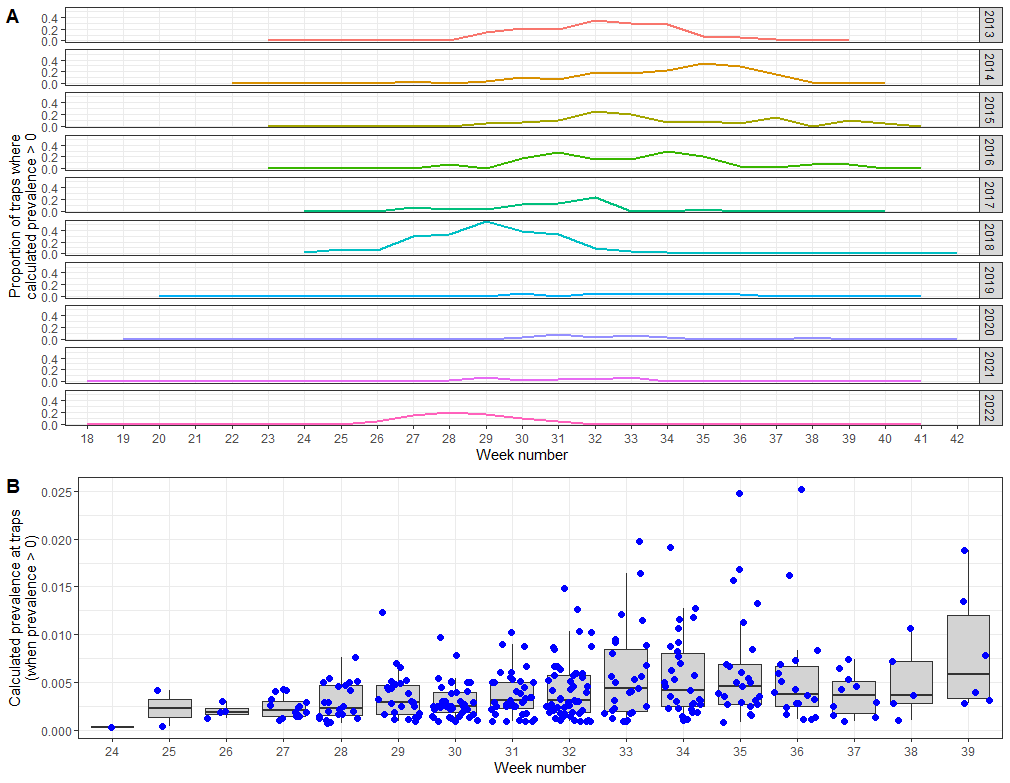

Supplement: S13 Fig — Proportion of traps where the calculated prevalence was greater than zero, per week and year (A) and the calculated prevalence when greater than zero per week number, grouped over the years, shown with data points in blue and boxplot summaries in grey (B). (PNG) [file ppat.1013753.s017.png]

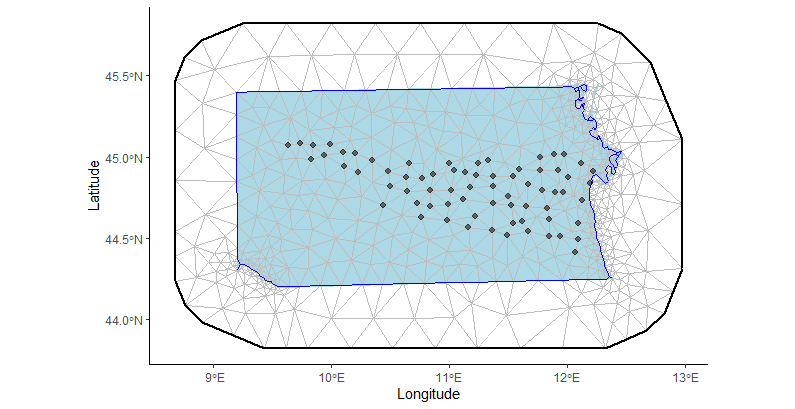

Supplement: S14 Fig — Emilia-Romagna area is shown in light blue, coordinates of the trap locations represented as black points, and the random field boundary in dark blue. The shapefile used to generate the administrative border was obtained from GADM (https://gadm.org/download_country.html and https://gadm.org/license.html). (TIFF) [file ppat.1013753.s018.tiff]

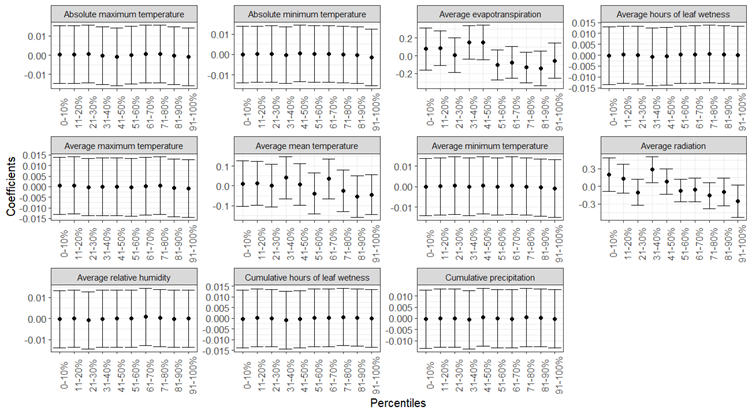

Supplement: S15 Fig — The meteorological variables were calculated at time lag = 0 (in the week of mosquito trapping). (PNG) [file ppat.1013753.s019.png]

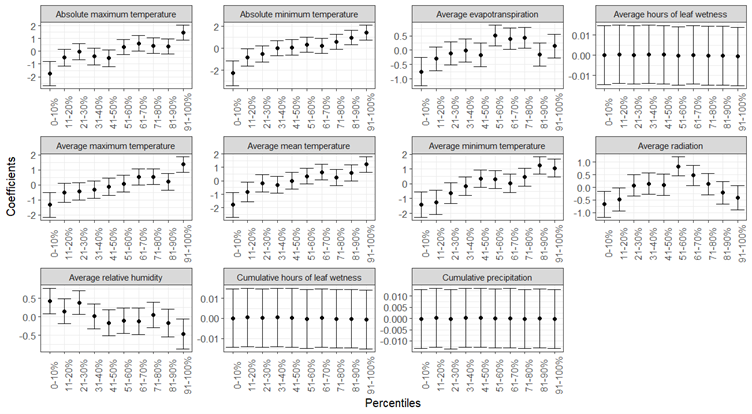

Supplement: S16 Fig — The meteorological variables were calculated at time lag = 0 (in the week of mosquito trapping). (PNG) [file ppat.1013753.s020.png]
